# Complete genome sequence of *Pseudomonas moorei* strain m318 isolated from rhizosphere of *Pteris multifida* in high-arsenic-content soil

**DOI:** 10.1128/mra.00774-24

**Published:** 2024-10-31

**Authors:** Ning Han, ChongYang Yang, Ying-Ning Ho, Ryota Moriuchi, Che-Chun Chen, Chihiro Inoue, Mei-Fang Chien

**Affiliations:** 1 Graduate School of Environmental Studies (GSES), Tohoku University, Sendai, Japan; 2 BioResource Research Center, RIKEN, Tsukuba, Japan; 3 Institute of Marine Biology and Center of Excellence for the Oceans, National Taiwan Ocean University, Keelung, Taiwan; 4 Taiwan Ocean Genome Center, National Taiwan Ocean University, Keelung, Taiwan; 5 Shizuoka Instrumental Analysis Center, Shizuoka University, Shizuoka, Japan; 6 Doctoral Degree Program in Marine Biotechnology, National Taiwan Ocean University, Keelung, Taiwan; California State University San Marcos, San Marcos, California, USA

**Keywords:** rhizobacterium, arsenic, plant-microbe interactions

## Abstract

*Pseudomonas moorei* strain m318 is an arsenite-oxidizing rhizobacterium isolated from the rhizosphere of an arsenic hyperaccumulator plant, beneficial for arsenic phytoremediation. Here, we report the complete genome sequence of this strain, which consists of a circular chromosome assembled using long reads sequenced on Nanopore and polished with Illumina paired-end reads.

## ANNOUNCEMENT

*Pseudomonas moorei* m318 (formerly *Pseudomonas vancouverensis* m318) was isolated from the rhizosphere of the arsenic hyperaccumulator *Pteris multifida* in high-arsenic-content soil, sampled in July 2019 in Japan (38°48′20.6″N 141°32′04.3″E). This strain shows high arsenic resistance and can rapidly oxidize arsenite to arsenate. Additionally, it produces various beneficial metabolites that enhance plant growth and nutrient acquisition (1). To better understand the functions of this promising microbial inoculant and its ability to promote phytoremediation efficiency at the genomic level, we report the complete genome sequence of *P. moorei* m318.

The bacterial strain was acquired as described in our previous study (1). In brief, the rhizospheric soil of *P. multifida* was diluted with TE buffer and spread on 0.2 × tryptic soy broth (TSB) agar plates containing 1 mM NaAsO_2_ or Na_2_HAsO_4_ at 30°C overnight. Colonies were selected and re-cultured in 0.2 × TSB medium for further investigation and stored in 25% glycerol at −80°C. A 100 µL of the glycerol-preserved culture was inoculated into 5 mL of 0.2 × TSB medium and cultured for 16 hours at 30°C with shaking at 150 rpm. Genomic DNA was extracted using the Wizard genomic DNA purification kit (Promega Co., MI, USA), following the manufacturer’s protocol, and 200 ng of the extracted DNA was used for subsequent sequencing.

The Illumina sequencing library was prepared using the Illumina TruSeq DNA PCR-free kit, and sequencing was performed on the Illumina MiSeq platform (2 × 300 bp, 600 cycles), generating a total of 3,089,390 reads and 926.9 Mbp of sequencing data. Long-read sequencing was performed by preparing Nanopore sequencing libraries with the ligation kit (SQK-LSK109) following the manufacturer’s instructions (Oxford Nanopore Technologies, Oxford, UK). Size selection was performed using AMPure beads, removing fragments smaller than 1,000 bp. Nanopore sequencing was conducted for 96 hours on the MinION platform using flow cell R9.4.1, producing 45,504 reads with an *N*_50_ read length of 14,935 bp, totaling 231.8 Mbp. Quality control of the raw sequencing data from the Illumina and Nanopore platforms was conducted using fastp version 0.23.4 (2) and NanoPlot version 1.41.0 (3), respectively.

The long-read sequencing data obtained from the Nanopore was used for *de novo* genome assembly with Flye version 2.9.2 (4), resulting in a single circular contig with an average coverage of 35×. The contig was then polished using Medaka version 1.10.0 (Oxford Nanopore Technologies Ltd.). Subsequently, error correction and further polishing were performed using Pilon version 1.24 (5) with Illumina short reads. The genome assembly had a completeness of 100% and a contamination level of 0.37%, as confirmed by CheckM version 1.2.2 (6). The assembled genome consists of a single contig of 6,533,399 bp with a G+C content of 59.6%. The genome was annotated with DFAST version 1.3.1 (7), revealing 19 rRNA genes, 72 tRNA genes, and 6,053 protein-coding sequences. Strain m318 was confirmed as *Pseudomonas moorei* with FastANI version 1.3.2 (8) sharing over 98.16% average nucleotide identity with the reference strain CCUG 53114 (accession number GCA_008801475.1). Default parameters were used for all software.

## Data Availability

This genome project is indexed at DDBJ under BioProject accession number PRJDB18215. The Illumina raw reads are available at DRR598777, and nanopore raw reads are available at DRR574467. The assembly and annotation of this genome can be found under the accession number AP035776.
